# Evaluation of Recovery Time and Quality After Two Different Post-Operative Doses of Medetomidine in Spanish Purebred Horses Anaesthetized with Medetomidine–Isoflurane Partial Intravenous Anaesthesia

**DOI:** 10.3390/ani14223308

**Published:** 2024-11-18

**Authors:** Francisco Medina-Bautista, Juan Morgaz, Juan Manuel Domínguez, Rocío Navarrete-Calvo, Antonia Sánchez de Medina, Setefilla Quirós-Carmona, María del Mar Granados

**Affiliations:** Section Anaesthesiology, Department of Animal Medicine and Surgery, Veterinary Teaching Hospital, University of Córdoba, 14014 Córdoba, Spain; v92moroj@uco.es (J.M.); nacar6@hotmail.com (R.N.-C.); tsmedina189@hotmail.com (A.S.d.M.); setequica@hotmail.com (S.Q.-C.); pv2grmam@uco.es (M.d.M.G.)

**Keywords:** anaesthesia, horses, medetomidine, quality, recovery, time

## Abstract

Recovery from general anaesthesia is risky in horses. Alpha_2_-agonist administration after anaesthesia enhances the quality of recovery but may prolong this phase. Recovery time and quality were investigated after medetomidine administration at the end of general anaesthesia in a prospective, randomised, masked and clinical study. Horses underwent medetomidine–isoflurane partial intravenous anaesthesia. Medetomidine (0.5 or 1 µg/kg) IV was administered just after isoflurane was discontinued. The duration of different recovery phases and the number of attempts were recorded. A composite scale (from 1—excellent to 6—accident) was used for quality assessment. Mann—Whitney U-test was performed (*p* < 0.05). Twenty-seven horses per group were included. Results for 0.5 and 1 µg/kg groups were as follows: lateral recumbency time: 35 (24–45) and 43 (35–55) minutes; sternal recumbency time: 6 (3–15) and 5 (2–15) minutes; total recovery time: 47 (40–59) and 49 (42–62) minutes; number of attempts to sternal: 1 (1–1) and 1 (1–2) and to standing: 1 (1–3) and 1 (1–2); and, quality: 2 (1–2) and 2 (1–2), respectively. No significant differences between the groups were found. Medetomidine 0.5 µg/kg dose did not decrease the recovery time but maintained the recovery quality.

## Simple Summary

The mortality rate in horses undergoing general anaesthesia (GA) is 1%, which is higher than in small animals. This mortality rate is impacted by complications that occur during the recovery period, making it the riskiest phase in equine anaesthesia. It has been shown that using a postanaesthetic intravenous bolus dose of an α_2_-agonist improves recovery quality and increases its time. However, recovery time should not be prolonged as complications such as neuro/myopathy or hypoxaemia can occur. The objective of this study was to compare the recovery quality and time after administering two different doses of medetomidine at the end of the anaesthesia (0.5 or 1 µg/kg IV). There were no significant differences between the groups for recovery quality, recovery time, or the number of attempts to return to the standing position. It can be concluded that the low dose medetomidine postanaesthetic bolus (0.5 µg/kg IV) did not decrease the recovery time but maintained recovery quality when compared to the high dose medetomidine (1 µg/kg IV). Further research is necessary to optimise the quality–time ratio of recovery. A 50% reduction in the post-anaesthetic bolus dose of medetomidine was not enough to reduce the recovery time. Further studies using lower doses to reduce the time and maintain quality could be postulated.

## 1. Introduction

General anaesthesia (GA)-induced mortality rates are significantly higher in horses (1%) than in humans (0.000004–0.0007%), cats (0.24%), and dogs (0.14%) [1,2,3,4,5]. The mortality rate remains high (0.6%) even in horses administered anaesthesia for elective procedures [1].

Most deaths occur during recovery, between 81% and 100%, according to different sources, making recovery the most dangerous phase of anaesthesia [1,2,3,4,5,6]. Even in healthy patients undergoing non-colic procedures, half of the deaths happened during this phase [1]. Recovery can be assisted (manual or using ropes) or unassisted (free). In horses undergoing partial intravenous anaesthesia (PIVA), recoveries are free in 50% of cases and 45.1% assisted with ropes [1]. These data reflect the controversy about which is the best method of recovery [7,8,9,10]. The quality and duration of recovery are difficult to forecast due to their multifactorial nature, which may be affected by the invasiveness of surgery, anaesthesia time, body position, drugs and their doses, hypotension, and intrinsic factors, including temperament, age, and body mass [11,12,13,14,15,16].

Inhalational anaesthesia is often used in horses during GA, and it is associated with rapid recovery [17]. However, it leads to greater excitement and a higher number of attempts to stand, which can worsen the quality of recovery [12]. To improve recovery quality, a combination of two strategies is used (1) PIVA using continuous rate infusions (CRI) of an α_2_-agonist combined with isoflurane reduces the isoflurane requirements, with a consequent improvement in the recovery quality and the level of analgesia [18,19], and (2) post-GA administration of an α_2_-agonist bolus to prolong the recovery time, thus improving the quality of recovery [11,20,21,22,23].

Although studies have investigated the role of different α_2_-agonists to optimise the quality/time ratio of recovery, no definitive evidence regarding the superiority of any single α_2_-agonist has been reported [24]. Partial intravenous anaesthesia using a medetomidine CRI at 3.5 µg/kg/h with inhaled anaesthesia followed by a postanaesthetic bolus of medetomidine at 2 µg/kg reportedly results in good recovery quality [25,26]. However, medetomidine was associated with a poorer recovery quality when compared with equipotent CRIs and postanaesthetic doses of dexmedetomidine and longer recovery times when compared with xylazine [27]. Optimisation of the recovery period includes shortening recovery times, thereby minimising several associated complications whilst maintaining the quality of recovery [28].

In this study, we aimed to compare the time and quality of anaesthesia recovery after bolus administration of two different medetomidine doses (0.5 vs. 1 µg/kg) in Spanish Purebred horses that underwent arthroscopic surgery using medetomidine–isoflurane PIVA. We hypothesised that low dose medetomidine may shorten the anaesthesia recovery time while maintaining its quality.

## 2. Materials and Methods

### 2.1. Study Design

This prospective, randomised, masked clinical study was approved by the appropriate ethics committee (CEBAHCV 46/2020). Written informed consent was obtained from all horse owners.

Food, but not water, was withheld for 8 to 12 h prior to anaesthesia. Horses were randomly assigned to either the 0.5 M (medetomidine 0.5 µg/kg intravenous (IV)) or the 1 M (medetomidine 1 µg/kg IV) group based on a paper taken from a box containing 54 papers indicating the dose to give (27 papers each labelled as 0.5 M or 1 M), by an anaesthetist who did not participate in the recovery assessment. All anaesthetic procedures were performed by the same experienced anaesthetists (R.N-C., A.S.M, S.Q-C. or M.M.G.).

### 2.2. Animals

Fifty-four horses that presented to the Veterinary Teaching Hospital of Córdoba between November 2020 and May 2022 for arthroscopic surgery were included. The inclusion criteria were body weight > 200 kg, age between 2 and 20 years, American Society of Anaesthesiologists (ASA) physical status II, and unassisted recovery. The exclusion criteria were indications for assisted recovery, intraoperative changes in the medetomidine CRI rate or administration of GA in the previous 14 weeks [29].

### 2.3. Anaesthesia

The skin over the jugular vein was clipped and aseptically prepared before administrating the sedative agent. A 14G catheter (Equivet, KRUUSE, Langeskov, Denmark) was placed after a subcutaneous infiltration of 4 mL mepivacaine 2% (Mepivacaína 2%, BBraun, Barcelona, Spain). The animals in both groups received Na^+^ penicillin 22,000 I.U./kg IV (Penilevel, Laboratories Calier SA, Barcelona, Spain), gentamicin 6.6 mg/kg IV (Genta-Equine, Dechra, Barcelona, Spain), and phenylbutazone 4 mg/kg IV (Butasyl, Zoetis, Madrid, Spain) 30 min before the administration of premedication. Additionally, the horses were premedicated using medetomidine 7 µg/kg IV (Sedastart, BBraun, Barcelona, Spain) diluted to a total volume of 20 mL, and the depth of sedation was assessed 10 min later based on the criteria published by Taylor et al. [20]. Sedation was evidenced by the head height under the withers, an atonic lower lip, and no reaction to snapping fingers. In the absence of one criterion, a supplemental dose of medetomidine 2 µg/kg was administered, and sedation was reassessed 3 min later; this procedure was repeated if needed. Diazepam 0.1 mg/kg IV (Valium, Roche Farma SA, Madrid, Spain) and ketamine 2.5 mg/kg (Imalgene, Merial, Toulouse, France) were used to induce anaesthesia, and additional boluses of ketamine (0.5 mg/kg) were administered, if necessary, until intubation was achieved. Head and tail ropes were used to assist in the induction of general anaesthesia. The trachea was intubated using a silicone endotracheal tube (ETT, internal diameter 26–30 mm). Horses were transferred to a padded surgery table (Equine Operating Table Haico Telgte II, Eickemeyer, Tuttlingen, Germany), positioned in dorsal recumbency, and connected to a large animal circle system (LDS 3000 Large Animal Anesthesia Machine, Surgivet, Smith Medical, Barcelona, Spain). Volume control mechanical ventilation was initiated to maintain normocapnia (tidal volume 10–12 mL/kg, respiratory rate 6–8 breaths/min, and airway pressure 20–25 cmH_2_O) in all horses. The capnometer and gas analyser were calibrated before this study started. Anaesthesia was maintained with isoflurane (IsoVet, Piramal Healthcare UK Limited, Northumberland, UK) vaporised in 100% oxygen and a CRI of medetomidine at 3.5 µg/kg/h. Expiratory isoflurane concentration (FE’Iso) was maintained within a range between 1% and 1.2%. The depth of anaesthesia was assessed at 5 min intervals, and the isoflurane supply was readjusted based on the muscular tonus, detection of nystagmus, and palpebral reflex. Five minutes after starting mechanical ventilation, morphine 0.2 mg/kg IV (Morfina 2%, BBraun, Barcelona, Spain) was administered gradually for 2 min. Ringer’s lactate was administered (5 mL/kg/h) throughout anaesthesia, and a urinary catheter was placed in all horses. Heart rate, respiratory rate, end-expiratory carbon dioxide tension (PE’CO_2_), FE’Iso, and arterial blood pressures were monitored continuously using a multiparameter monitor S/5 (Datex Ohmeda, GE Healthcare, Helsinki, Finland) and collected as the mean over the whole anaesthesia time. An 18G catheter (Vasocan, BBraun, Barcelona Spain) was inserted percutaneously into the transverse facial artery to measure invasive arterial blood pressure. An extension line filled with heparinised saline solution was connected to the arterial catheter, and an electronic transducer (Clear-Cuff pressure infusor, Smiths Medical ASD Inc., Norwalk, CA, USA) was placed at the level of the manubrium sternum and zeroed to atmospheric pressure. Dobutamine (Dobutamina, Inibsa Hospital SLU, Barcelona, Spain) was initiated at a rate of 0.25 µg/kg/min via an infusion pump (NIKI v4 volumetric infusion pump, Grayline Medical, Norwalk, CA, USA) when the mean arterial pressure (MAP) was <70 mmHg. The dobutamine infusion rate was adjusted by increments of 0.25 µg/kg/min at 5 min intervals as necessary. A bolus dose of 0.5 mg/kg of ketamine IV was administered to horses that developed intense nystagmus, and animals that showed movement were administered thiopental IV (0.5 mg/kg) (Tiobarbital 1 g, BBraun, Barcelona, Spain).

Mechanical ventilation was stopped 5 min before the horse was transported to the recovery box. Horses that developed apnoea were manually ventilated until the return of spontaneous ventilation. Simultaneously, the arterial line was removed, and all monitoring devices were disconnected. Isoflurane and medetomidine CRI were maintained until the horses were transported to the recovery box, and a pre-recovery bolus of medetomidine 0.5 or 1 µg/kg diluted in saline to 10 mL total volume was administered just before disconnecting the horse from the anaesthesia machine.

### 2.4. Recovery

Horses were positioned in left lateral recumbency, with the carpal joints extended and the head, neck, and body aligned in the same plane within the recovery box. Their sweat was dried, the mouth was moistened, and oxygen flow-by was supplemented at 10 mL/kg through the ETT. The trachea was extubated following the observation of ear movement or nystagmus in the presence of a stable respiratory rate. If the presence of signs compatible with nasal oedema appeared, 5 mL of 0.15% phenylephrine was distributed into each nostril. The lights were dimmed, and the horses were left undisturbed in the recovery box. All recoveries were unassisted.

The anaesthetist who did not participate in any anaesthesia (F.M-B.) assessed the recovery quality in all horses. To assess recovery quality, a composite scoring system consisting of eight parameters (general attitude, movement to the sternal position, sternal phase, standing movement, strength of movements, number of attempts to stand, balance and coordination, and fetlock weakness during standing) was used. An individual score was recorded for each variable, and the sum was translated on a scale ranging from 1 (indicative of calm and easy recovery) to 6 (indicative of an accident) [30]. A subdivision was made inside this classification as previously described [31]; the recoveries with a score of 1 or 2 were considered acceptable recoveries, whereas those with a score of 3, 4, 5 or 6 were considered unacceptable recoveries.

Recovery times were recorded as follows: anaesthesia time (interval between anaesthesia induction and isoflurane discontinuation), surgery time (interval between the first incision and tying the last suture), extubation time (interval between pre-recovery bolus administration and endotracheal extubation), lateral recumbency (time extending between pre-recovery bolus administration and sternal position), sternal time (time between adopting the sternal and standing positions (understanding standing position when the animal could be safely approached)), and total recovery time (interval between pre-recovery bolus administration and the standing position). The number of attempts required to achieve the sternal and standing positions was also recorded.

### 2.5. Data Analysis

The sample size was previously calculated using the “total recovery time” variable. Considering the authors’ clinical experience in achieving good quality recoveries using medetomidine at 1 µg/kg IV, the standard deviation (SD) was calculated based on a pilot study in horses that received a 1 µg/kg dose. Considering a power of 0.8 and an alpha error of 0.05, 24 horses needed to be assigned to each group to detect intergroup differences of 10 min. A 10 min interval was selected based on the results of previous studies [25,26], which reported an interval of approximately 60 min between the administration of medetomidine at 2 µg/kg IV and standing. In our pilot study, the standing time after the administration of medetomidine at 1 µg/kg IV was 50 ± 12 min. Therefore, it was reasonable to conclude that the administration of 50% of the dose may reduce the recovery time by 10 min. Considering our study design (clinical study), a 10% loss to follow-up was expected, and finally, 27 horses were included in each group. Shapiro–Wilk test was used to determine the normality of variables. For normally distributed variables, a *t*-test was used to determine differences between the groups. For variables with a non-Gaussian distribution, a Yuen test was applied to compare quantitative variables between groups, with a trimming of 0.2. The Mann–Whitney U test was used for ordinal variables (recovery quality). Fisher’s exact test was used to confirm intergroup differences in breed, sex, number of operated limbs, number of operated joints, number of operated front and back limbs, and use of dobutamine (yes/no), ketamine (yes/no), or thiopental (yes/no). The IBM SPSS Statistics software version 28 (IBM, Armonk, NY, USA) was used to perform classical statistical analysis, and the function yuen included in the package WRS2 of R 4.1.2 (R Foundation for Statistical Computing, Vienna, Austria) was used to perform the robust Yuen test. Normally distributed variables are expressed as mean ± SD and nonnormally distributed quantitative and ordinal variables as median (P25th–P75th). Nominal variables are expressed as frequencies. Statistical significance was set at *p*-value < 0.05.

## 3. Results

**Figure 1:**
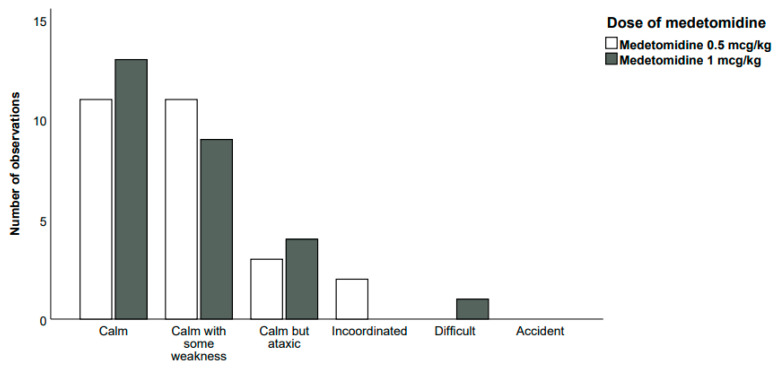
Recovery quality assessment according to the composite scoring system for both groups.

Fifty-four horses (27 horses in each group) were studied, and no horses were excluded. Demographic data (sex, age, body weight, breed, number of operated limbs, number of operated joints and number of operated front and back limbs) are represented in Table 1, showing no significant differences between the two groups. Regarding the intraoperative parameters (Table 2), no significant differences in anaesthesia or CRI duration were observed between the groups. Intergroup differences in intraoperative parameters were only observed in the FE’Iso, showing slightly higher values in the 1 M group than in the 0.5 M group (0.11%, 95% confidence interval 0.01–0.2%, *p* = 0.03; Cohen’s d = 0.122). Regarding recovery data (Table 3 and Figure 1), no significant intergroup differences were observed regarding recovery quality or any other recovery parameter.

Dobutamine administration was required in both groups; specifically, 74.1% (20/27) of horses received dobutamine, and no significant differences were detected between treatments (*p* = 0.999). Similarly, no significant differences were observed regarding ketamine administration (*p* = 0.610), with 7.4% (2/27) of horses in the 0.5 M group and 3.7% (1/27) in the 1 M group receiving one ketamine bolus. No animal in this study required thiopental.

## 4. Discussion

Our results indicated that recovery quality in horses that underwent arthroscopic surgery after the administration of a medetomidine bolus was acceptable regardless of the dose; however, a low dose (0.5 µg/kg) did not shorten the recovery time. Therefore, these findings partly support the hypotheses tested in this study.

The reasons why α_2_-agonist administration at the end of GA improves recovery quality may be the sedative and analgesic properties of these drugs. During recovery, anaesthetic gases such as isoflurane are detected in the expired air for at least 25 min [12]. Although these subanaesthetic concentrations do not prevent voluntary movement, they may cause neurological effects, such as cognitive function decline, loss of proprioception, impaired motor neuron function, and hyperalgesia [32,33,34,35]. Alpha_2_-agonist administration during recovery prevents futile attempts to stand [36] and results in a prolonged elimination of inhalational agents (anaesthetic lavage). Therefore, inhalational anaesthetics are progressively eliminated with a more coordinated recovery of neurological function [21]. In the present study, while the variable FE’Iso significantly differed between the groups (1 M: 1.2% vs. 0.5 M: 1.1%), these differences were not clinically relevant. Furthermore, no other intergroup difference was observed with regard to intraoperative parameters, especially for the use of ketamine or dobutamine, which may indicate a light or deep anaesthetic plane, respectively.

Improvement in recovery quality following α_2_-agonist administration may indicate prolonged recovery time [23]. However, this prolonged recovery may lead to detrimental effects owing to the high risk of myopathy, neuropathy, and hypoxaemia associated with prolonged lateral recumbency, especially when considering that the most represented breed in this study is characterised by its robust morphology [28,37,38]. In the present study, the administration of a medetomidine bolus led to good recovery quality with a recovery time of 47 (40–59) min and 49 (42–62) min in the 0.5 M and 1 M groups, respectively. These recovery times are similar to those observed after detomidine (at 2.5 and 5 µg/kg) or romifidine (at 20 µg/kg) administration under similar conditions (horses undergoing elective procedures, mostly arthroscopy, under PIVA), 42 ± 14, 42 ± 17 and 44 ± 15 min, respectively [34]. Notably, the 0.5 µg/kg dose did not shorten the recovery time. However, whether a further lowering of the dose reduces the recovery time requires further investigation. Kalin et al. (2021) [25] observed that the recovery time following medetomidine administration was longer than that recorded after xylazine administration; however, the medetomidine dose used was higher than that used in our study. Additionally, anaesthesia and consequent PIVA durations were longer in the previous study than in this study, and the recovery time could have increased by medetomidine accumulation. Recovery time for the medetomidine groups in our study was shorter than that reported by Kalin et al. (2021) [25] when xylazine was administered. Although the surgical procedure reported by Kalin et al. (2021) [25] was not standardised, and the horses in the medetomidine group underwent colic surgery, the same research group concluded that recovery time was shorter when xylazine was administered at the end of anaesthesia than in the medetomidine group in horses that were anaesthetised for elective procedures. However, the shorter time achieved with the xylazine group in that study by Wiederkehr et al. (2021) [26] was the same as that recorded in this study, and the recovery quality between the xylazine and medetomidine groups was unaffected.

Medetomidine has the same α_2_:α_1_ selectivity ratio as dexmedetomidine but is more selective for the α_2_ receptor than romifidine, xylazine, and detomidine [39]; however, it appears that this selectivity is not related to recovery quality. Detomidine, despite having a higher α_2_:α_1_ selectivity than xylazine, produced worse recoveries than xylazine [40], possibly due to more pronounced ataxia, and a similar finding was observed upon comparison with romifidine [41]. While medetomidine and dexmedetomidine have the same selectivity ratio, dexmedetomidine produced a better quality of recovery but did not shorten recovery times despite its rapid elimination [25]. Existing studies have reported conflicting results regarding recovery time after α_2_-agonist administration. Moreover, the time points used to establish different recovery times are not clearly defined in the literature. Further studies that investigate context-sensitive half-time in contrast to half-life are warranted to gain a deeper understanding of the variables mentioned above. Additionally, the use of α_2_-agonist CRIs alongside inhalational agents (PIVA) should be investigated to understand the impact this technique has on the quality and duration of recovery [42].

Although trials have reported the same reliability of results, regardless of the complexity and lack of a validated scale, we used a composite scoring system in this study. Compared with simpler scales, the composite scoring system considers most of the factors that impact recovery quality, thus reducing subjectivity [43,44]. In a study using a methodology and recovery scale similar to that used in this research, most recoveries obtained scores of 2 and 3, whereas the recovery quality was mostly 1 and 2 in the current study. Notably, the abovementioned study used postanaesthetic sedation of 2 μg/kg of medetomidine IV, thus implying that a higher dose does not necessarily equal better quality recoveries [26].

Several studies have investigated risk factors associated with recovery from GA in horses [15,45,46]. General anaesthesia administered to horses is known to be risky, with a mortality rate of 0.6% in horses that underwent elective procedures [1]. While the breed is a possible risk factor for mortality [21], the Spanish Purebred Horse was the most common breed included in this study. This breed is well known for its docility, which may have contributed to the good quality of the recoveries observed in our study [38].

Another limitation of this study is that the recovery quality was recorded by a single observer; however, the observer had considerable experience in this kind of procedure. Furthermore, as few studies have investigated the role of context-sensitive half-times of other drugs used during anaesthesia, it is difficult to determine the effects of postanaesthesia sedation [47].

## 5. Conclusions

Administration of a medetomidine bolus of 0.5 or 1 μg/kg IV prior to recovery of horses that were anaesthetised using a PIVA of isoflurane and a CRI of medetomidine (3.5 μg/kg/h) for arthroscopic surgery produced similar recovery quality and recovery times.

## Figures and Tables

**Table 1:** Demographic data (median (P25th–P75th)). Significance was considered when *p* < 0.05.

Variable	Medetomidine 0.5 µg/kg	Medetomidine 1 µg/kg	*p* Value
Sex (%)	77.8 S/22.8 M	85.2 S/14.8 M	0.728
Age (years)	4 (3–5)	4 (2–6)	0.911
Body weight (kg)	520 (488–540)	520 (456–570)	0.945
Breed (%)	70.4 SPH 18.5 LH 7.4 HSH 3.7 WBH	51.9 SPH 7.4 AAH 3.7 APH 7.4 SSH 14.8 WBH 11.1 LH 3.7 HAH	0.258
Number of operated limbs (%)	40.9 1L/59.1 2L	56.5 1 L/43.5 2 L	0.376
Number of operated joints	1.5 ± 0.6	1.7 ± 0.6	0.438
Position of operated limbs	3 FL/20 BL/4 FBL	5 FL/21 BL/1 FBL	0.423
Total extra medetomidine dose (µg/kg)	20	18	0.999
Total extra ketamine dose (mg/kg)	7	9.5	0.999

S: stallion; M: mare; SPH: Spanish Purebred horse; LH: Lusitan horse; HAH: Hispano-Arabian horse; WBH: Warmblood horse; AAH: Anglo-Arabian horse; APH: Arabian Purebred horse; SSH: Spanish Sport horse; 1L: one operated limb; 2L: two operated limbs; FL: front limbs surgically involved; BL: back limbs surgically involved; FBL: both, front and back limbs surgically involved.

**Table 2:** Values of intraoperative parameters (mean ± standard deviation or median (P25th–P75th)). Significance was considered when *p* < 0.05.

Variable	Medetomidine 0.5 µg/kg	Medetomidine 1 µg/kg	*p* Value
CRI medetomidine time (min)	85 (60–90)	75 (55–95)	0.743
Anaesthesia time (min)	95 (77–110)	90 (70–116)	0.595
Surgery time (min)	55 (35–65)	50 (40–65)	0.772
HR (beat per min)	34 ± 4	34 ± 5	0.855
RR (breath per min)	7 (6–7)	6 (6–7)	0.187
SAP (mmHg)	108 ± 8	107 ± 8	0.907
MAP (mmHg)	81 ± 9	83 ± 7	0.294
DAP (mmHg)	66 ± 7	69 ± 5	0.098
PE’CO_2_ (mmHg)	42 ± 2	42 ± 3	0.961
FE’Iso (%)	1.1 (1–1.2)	1.2 (1.1–1.3)	0.03 *
Dobutamine amount (mg/kg)	10 (0–18)	9 (0–16)	0.825
Dobutamine time (min)	30 (0–50)	35 (0–60)	0.77

* Denotes significant statistical differences between groups. CRI: constant rate infusion; HR: heart rate; RR: respiratory rate; SAP: systolic arterial pressure; MAP: mean arterial pressure; DAP: diastolic arterial pressure; PE’CO_2_: end-expiratory carbon dioxide tension; FE’Iso: expiratory isoflurane concentration.

**Table 3:** Values of recovery parameters (mean ± standard deviation or median (P25th–P75th)). Significance was considered when *p* < 0.05.

Variable	Medetomidine 0.5 µg/kg	Medetomidine 1 µg/kg	*p* Value
Lateral recumbency time (min)	35 (24–45)	43 (35–55)	0.062
Sternal time (min)	6 (3–15)	5 (2–15)	0.836
Sternal attempts	1 (1–1)	1 (1–2)	0.237
Total recovery time (min)	47 (40–59)	49 (42–62)	0.507
Standing attempts	1 (1–3)	1 (1–2)	0.214
Extubation time (min)	8.1 ± 3.7	9.0 ± 4.7	0.433
Composite scoring system	2 (1–2)	2 (1–2)	0.661
Recovery acceptability	22 A/5 NA	22 A/5 NA	0.735

A: acceptable recovery; NA: unacceptable recovery.

## Data Availability

Data are available at https://github.com/v52mebaf/Medetomidine-Animals-Base-data.git (accessed on 17 December 2023).
